# Self-monitoring of blood pressure for preeclampsia patients: Knowledge and attitudes

**DOI:** 10.4102/curationis.v44i1.2195

**Published:** 2021-09-21

**Authors:** Johanna Munyungula, Simangele Shakwane

**Affiliations:** 1 Department of Health Studies, College of Human Sciences, University of South Africa, Pretoria, South Africa; 2 Council of Medical Schemes, Pretoria, South Africa

**Keywords:** blood pressure, patients, preeclampsia, pregnant, self-monitoring

## Abstract

**Background:**

Preeclampsia is one of the causes of maternal deaths and is also responsible for complications such as premature births worldwide. In South Africa, hypertensive disorders cause 14% of all maternal deaths. Evidence indicates that it may be beneficial to empower women to monitor their blood pressure (BP) in the comfort of their homes.

**Objectives:**

The purpose of this study was to explore and describe preeclampsia patients’ knowledge and attitudes towards the self-monitoring of their BP.

**Method:**

An exploratory, descriptive and contextual qualitative research study was conducted. Fourteen preeclampsia patients were purposively sampled and participated in the study. In-depth semi-structured interviews were used to collect data. Data were analysed using the thematic analytic approach.

**Results:**

The knowledge and attitudes towards the self-monitoring of blood pressure (SMBP) were explored. Four themes emerged, namely understanding of hypertension disorders during pregnancy, openness on self-monitoring at home, its hindrances and benefits. The participants portrayed limited understanding and knowledge of preeclampsia, yet they had positive attitudes towards monitoring BP themselves and were open and willing to do self-monitoring at home.

**Conclusion:**

The use of SMBP may relieve overcrowding in public healthcare institutions. Encouraging patients to participate in self-monitoring could promote active participation and a positive outlook on their pregnancies. The unavailability and unaffordability of the equipment may pose a challenge to women with a low socioeconomic status.

## Introduction

Worldwide, 10% of pregnancies are complicated by hypertensive disorders such as preeclampsia, which is one of the most important causes of maternal and perinatal morbidity and mortality (Say et al. 2014:e323). In 2017, the two regions namely sub-Saharan Africa (SSA) and Southern Asia were responsible for 86% (254 000) of the global maternal deaths, with SSA contributing nearly 66% of the deaths (196 000) (World Health Organization [WHO] 2019). Preeclampsia is distinguished by high blood pressure (BP), with protein in the urine that generally develops after the 20th week of pregnancy (Preeclampsia Foundation 2018). As the exact cause of preeclampsia is unknown, one specific treatment for it is also unknown (National Department of Health [NDoH] 2015:12).

The Saving Mothers 2014–2016 report indicates that in South Africa, there has been a slight improvement in the quality of care, as seen in the slow but steady decline in the number of potentially preventable maternal deaths. However, there was no change in mortality related to hypertensive diseases in pregnancy; instead, the country has experienced an increase of 14% (Department of Health 2018:8). A knowledge deficit regarding preeclampsia has been reported to attribute to the failure in the improvement and management of the condition (Maputle, Khoza & Lebese 2017:47). Primary preventative care is of paramount importance for the early detection and management of hypertension disorders in pregnancy (Salomon et al. 2019:411; Tucker et al. 2017:442).

Self-monitoring of blood pressure (SMBP) is the measuring of BP by individuals in the comfort of their home environment. The SMBP values or results may be lower than clinic BP measurements (Metoki et al. 2017:107). The study of Maputle et al. (2017:47) states that SMBP may decrease the BP measurement though with a small margin but significantly when compared to the clinic environment. Self-monitoring might reduce healthcare costs by reducing the number of clinic visits (Hodgkinson et al. 2014:349). In the present era of the COVID-19 pandemic, SMBP may assist clinics in reducing the overcrowding of patients for BP check-ups and movement of people in the community, thus, maintaining social distancing. Currently, limited data exist regarding SMBP in pregnancy for preeclampsia patients in resource-restricted communities; therefore, there is a need for healthcare practitioners and preeclampsia patients to work together to manage and prevent complications by employing self-monitoring at home.

This study aimed to explore and describe preeclampsia patients’ knowledge and attitudes towards the self-monitoring of their BP. The study had three objectives, which were:

- To explore the knowledge of preeclampsia patients about the use of a digital BP monitoring machine at home.
- To explore and describe the attitudes of preeclampsia patients towards SMBP.
- To identify the benefits and challenges of SMBP amongst preeclampsia pregnant women.

## The research method and design

### Study design

A generic qualitative research approach, which is exploratory, descriptive and contextual in nature (Creswell 2013:45; Polit & Beck 2017:12), was employed. This allowed the researcher to collect data in the natural setting to explore and describe the knowledge and attitudes of preeclampsia women towards SMBP. The constructivism paradigm was applied to construct knowledge through social interactions. The qualitative research design and constructivism paradigms complemented each other by relying upon the participants’ views and their understanding of the situation being studied (Creswell 2013:45) and acknowledging the impact of their past experiences. The participants developed subjective meanings of their own experiences and knowledge of the subject under study that led to the researchers generating individual perspectives of meanings into several categories or themes and ideas (Creswell 2013:46). The researchers worked collaboratively with participants and obtained data by asking open-ended questions to encourage them to explain their understanding of and views about the subject under discussion.

### Setting

The study was conducted in Gauteng province, in one of the public hospitals in the Tshwane Health District region. The selected hospital’s antenatal clinic (ANC) renders healthcare services to high-risk pregnant women. The patients attending the ANC are referred from primary healthcare clinics and community health centres in the catchment areas.

### Study population and sampling strategy

The population was sampled from pregnant women diagnosed with preeclampsia, who voluntarily consented to participate in the study. A non-probability sampling approach was used. A total number of 14 patients attending ANC were sampled using the purposive sampling method. This sampling technique allowed the researcher to make specific choices about which patients possessed a wealth of information about their personal experience about the condition to include in the research sample (Bertram & Christiansen 2014:60; Gray, Grove & Sutherland 2017:345). During the data collection period, the researchers went to the clinic in the mornings and selected the files of patients diagnosed and treated for preeclampsia.

The files were checked to select patients who met the inclusion criteria for the study, which were patients aged 18 years and above, a patient diagnosed with preeclampsia and attending ANC. The study excluded hospitalised patients and those aged below 18 years.

### Trustworthiness

The trustworthiness of the study was ensured using the components and criteria of Lincoln and Guba (1985). To ensure credibility, the researcher collected data and engaged with participants in the natural setting. Data were collected using semi-structured interviews and unstructured observations. Field notes were taken during interviews, and interviews were audio-recorded for the accuracy of information. Transferability was ensured using an interview guide to collect relevant data, and thick descriptions of the findings were presented. To ensure the reliability of data, verbatim transcriptions were conducted, and the services of an independent co-coder were used to analyse the data. The researchers avoided bias by maintaining transparency using the research trail, where the research process and activities were documented.

### Data collection method

The researchers provided information to the patients who met the inclusion criteria. The overview of the study was explained, including the purpose, data collection procedure, voluntary participation and audio recording during interviews to allow participants to make an informed decision. Participants signed informed consent before participating in semi-structured interviews that were recorded. Participants were assured of confidentiality and anonymity. They were advised of their right not to answer questions they felt uncomfortable with, and they were free to withdraw at any time from the interview. Semi-structured interviews were conducted between 4 September 2018 and 10 September 2018, and data were collected from 14 participants. Data saturation was reached after 12 interviews; the researchers conducted two more interviews to confirm that no new information was attained. Three participants were interviewed per day and interviews lasted for 30 min – 40 min. An interview guide was used with open-ended questions, which allowed the participants to describe their knowledge and attitudes towards SMBP and the researchers to ask follow-up probing questions when more information was required. Box 1 displays the questions that were asked during the interviews.

BOX 1 Interview guide.

**Table:** 

**Semi-structured questions:**
What is your understanding of high blood pressure in pregnancy?
Would you share with me your views about self-monitoring of blood pressure at home?
What would be the benefits of self-monitoring of blood pressure at home?
What are the challenges that can prevent you from monitoring your blood pressure at home?

After demographic information regarding age, number of pregnancy, gestational age, employment history and preeclampsia history, the interview guide questions (Box 1) were posed to all the participants.

### Ethical considerations

The researcher received ethical clearance from the University of South Africa Research Ethics Committee. Approval to conduct the study was obtained from the Department of Health and the selected hospital in Tshwane. Participants were provided with detailed information about the study, and they voluntarily signed informed consent forms to participate in the study. Exclusion from the study was based on the set inclusion criteria. Participants’ information was kept private using codes for all interviews and the co-coder signed the confidential agreement. Participants were informed that they had the freedom to withdraw from the study at any time.

## Results

### Demographic results

The age of participants ranged from 18 to 43 years. Four (28.6%) participants were at an advanced maternal age, which is a pregnancy that occurs at 35 years or older (Dekker, Niles & Breakey 2016:9) and 10 (75.4%) participants were aged between 19 and 34 years. The gravida results reflected that three (21.4%) of the participants were primigravidae (pregnant for the first time), whereas 11 (78.5%) were multiparous women (having been pregnant before). Eleven (11) participants have received education up to high school level, one achieved a tertiary qualification, and two never attended school. Seven (50.0%) of participants were employed, and the other seven (50.0%) were unemployed and depend on social grants for a living.

### Semi-structured interview results

The main aim of the study was to explore and describe preeclampsia patients’ attitudes towards the self-monitoring of their BP. The findings of the complete research study reflected in four themes, and the relevant sub-themes are summarised in Table 1. The themes and sub-themes represented the knowledge and attitudes of preeclampsia patients towards SMBP that emerged during the data analysis of this study.

**TABLE 1:** Themes and sub-themes.

Themes	Sub-themes
**Theme 1:** Understanding of hypertension disorders during pregnancy	Limited information onBP in pregnancy. Knowledge of the causes, effects and management of high BP during pregnancy.
**Theme 2:** Openness to SMBP at home	Willingness to SMBP at home. Reluctance to SMBP at home.
**Theme 3:** Hindrances in SMBP	Affordability and availability of the BP measuring machine. Lack of self-confidence and knowledge regarding the use of the BP measuring machine.
**Theme 4:** Benefits of using SMBP	Early identification of abnormalities and seeking medical attention urgently. Safety of the mother and the unborn baby. BP, blood pressure; SMBP, Self-monitoring of blood pressure.

### Theme 1: Understanding of hypertension disorders in pregnancy

Knowledge of BP is of particular importance for preeclampsia patients. In the discussion during the interview, the participants reflected their limited understanding of BP in pregnancy, as well as its causes and management. The sub-themes that give insight into these themes are discussed below.

**Limited information about high blood pressure in pregnancy:** The majority of the participants indicated that during their attendance in the healthcare facility, they did not receive any information about BP in pregnancy. In contrast, a few participants admitted having received limited information about preeclampsia from the doctor and other healthcare workers such as nurses. Little knowledge was expressed by the following quotes:

‘… Even now I don’t have a full understanding … because, no one has ever explained to me, I just read.’ (P06, 29 years, 5 Sept 2018)

‘… I don’t understand anything about it. Because I didn’t even have it before. I don’t know anything about it.’ (P13, 43 years, 10 Sept 2018)

‘Blood pressure, according to me, we were never taught about it … on the baby … I don’t know what causes it …’ (P12, 19 years, 7 Sept 2018)

A few participants indicated hearing about the condition from healthcare practitioners during a consultation and said that:

‘… I once heard the doctor saying if you have high blood pressure … you deliver a baby when you are thirty, thirty-six or I don’t know… I am not sure?’ (P10, 20 years, 7 Sept 2018)

‘I hear them [*nurses*] saying it is dangerous. It is dangerous when it is high. What it does, for what I have seen some end up having children early because it is dangerous when it is too high …’ (P02, 37 years, 4 Sept 2018)

**Knowledge of the causes, effects and management of high blood pressure during pregnancy:** The majority of the participants understood the general causes of high BP as associated with dietary lifestyles and psychological problems such as stress. To control high BP, participants believed in eating healthy, exercising and taking prescribed medication. This understanding is depicted by the following quotes:

‘[… *A*]ccording to me I think it is caused by food high in salt … and fats and when the heart beats fast. That’s how I understand it …’ (P12, 19 years, 7 Sept 2018)

‘Listen, high blood pressure is caused by stress or when worrying too much.’ (P03, 22 years, 4 Sept 2018)

‘… I can say it is stress. And maybe to worry a lot?’ (P07, 41 years, 6 Sept 2018)

Most of the participants had basic insight into how to manage high BP. They focused on diet, exercise and taking prescribed medication. They discussed the management of hypertension in pregnancy as follows:

‘… You must exercise … eat right, reduce salt …’ (P06, 29 years, 5 Sept 2018)

‘… I shouldn’t eat salty food … I must take medication every day.’ (P12, 19 years, 7 Sept 2021)

‘… Eating veggies, your veggies reduce salt and mmm, fatty foods …’ (P05, 32 years, 5 Sept 2018)

Knowing how to manage high BP does not mean that patients are compliant with it; some patients acknowledged that they do not take their medication as they are supposed to, do not eat healthy foods and do not exercise.

‘[… *S*]ometimes you don’t take medication the way they told you to, you do skip at times …’ (P09, 30 years, 6 Sept 2018)

‘[… *S*]ome of us don’t exercise …’ (P07, 41 years, 6 Sept 2018)

‘[… *L*]ack of exercise … due to extreme tiredness.’ (P11, 30 years, 7 Sept 2018)

‘… I heard that food high in fats and salt … but I do eat salty foods such as …’ (P10, 20 years, 7 Sept 2018)

All the participants were aware of the effects or complications of uncontrolled high BP in pregnancy, and they reported their knowledge of the effects on/complications for both the mother and the baby as follows:

‘I hear them saying it is dangerous. It is dangerous when it is high. What it does, for what I have seen some end up having children early because it is dangerous when it is too high …’ (P02, 37 years, 4 Sept 2018)

‘… It can kill the mother and the baby easily…’ (P01, 35 years, 4 Sept 2018)

‘To me, it can cause seizures and I end up having a stroke … Or if it’s too high, it may go to heart attack …’ (P06, 29 years, 5 Sept 2018)

‘It can affect both the mother and the baby. Maybe suffer from stroke … you end up with miscarriage …’ (P03, 31 years, 4 Sept 2018)

‘… Even a child can be born with abnormalities …’ (P07, 41 years, 6 Sept 2018)

Based on the discussion about their knowledge of high BP in pregnancy, a few participants portrayed limited knowledge about high BP in pregnancy that they received from healthcare workers such as doctors and nurses. In contrast, the majority reported having never received such information. This suggests that healthcare practitioners working in ANCs do not provide pregnant women with the necessary information about hypertension in pregnancy.

### Theme 2: Openness to self-monitoring of blood pressure at home

The majority of participants showed interest and openness to SMBP at home; however, a few acknowledged challenges. The participants’ views are discussed in the relevant sub-themes below.

**Willingness to self-monitoring of blood pressure at home:** The majority of the participants admitted that SMBP at home is a good thing to do and more acceptable than more frequent clinic visits. Some also stated that it is still essential to check BP at home to get to know what is happening with the baby:

‘… Then, if you test and find it like this, so … it motivates you as you will know what is happening inside you …’ (P08, 33 years, 6 Sept 2018)

‘… I think it is the right thing … I will benefit lots of things because … Cause, I know that if I do check myself when I check myself at home …’ (P07, 41 years, 6 Sept 2018)

‘[… *T*]o check at home … I see it to be right because [*it will help*] so that I monitor my health …’ (P14, 33 years, 10 Sept 2018)

The majority of participants support SMBP because they believed that the home environment is private and relaxed. Participants were of the view that checking BP would make them aware of the high readings and thus, it would encourage them to seek medical help from the doctor or the clinic. Few participants indicated that monitoring at home would also help to reduce the frequent number of antenatal visits at the clinic, especially for those who attend the clinic only to check their BP.

**Reluctance to self-monitoring of blood pressure at home:** Few of the participants were reluctant and uncomfortable to do self-monitoring at home as they fear mistakes because they are unfamiliar with the machine. They were worried about inaccurate measurements and mentioned that it is better to go to the clinic because they can question the health professionals about their condition and clear their doubts. Participants do not feel safe to monitor at home because they believe anything wrong could happen to the mother and baby at home, so it is better to be monitored at the hospital and the clinic. These fears are supported by the following statements made by the participants:

‘… As a pregnant woman who is a risk … For me the way I see it … it is risky, because mmm, it feels not safe … it is better to monitor at a hospital …’ (P01, 35 years, 4 Sept 2018)

‘… I see this one at home, is not ok. It’s better to check at the clinic because nurses will help …’ (P14, 33 years, 10 Sept 2018)

‘I don’t understand the manner of self-monitoring because, I am sure that I won’t be able to check myself why? Because I don’t have the machine …’ (P10, 20 years, 7 Sept 2018)

The information above has depicted the willingness of participants to SMBP at home; although some reluctance was also noted on account of the fear of making mistakes because of unfamiliarity with the BP measuring machine.

### Theme 3: Hindrances in self-monitoring of blood pressure

Successful monitoring of BP at home requires preeclampsia patients to have access to or own the digital BP machine.

**Affordability and availability to buy the blood pressure measuring machine :** Preeclampsia patients will need to have an affordable BP machine to be able to check BP at home. The socio-economic status of the participants may make it difficult for them to acquire a reliable BP machine. Participants expressed themselves as follows:

‘I don’t understand the manner of self-monitoring because I am sure that I won’t be able to check myself why? Because I don’t have the machine …’ (P13, 43 years, 10 Sept 2018)

‘[… *T*]o check at home … you will find that others don’t have money to buy the monitor …’ (P09, 30 years, 6 Sept 2018)

‘[… *I*]f they show me how to use the monitor because … if I can get someone to provide me with the machine and show me the way how to use the monitor …’ (P02, 37 years, 4 Sept 2018)

**Lack of self-confidence and knowledg e on the use of the blood pressure machine:** Few of the participants were concerned about not knowing how to use the machine. They were also afraid that the device might show inaccurate measurements and put their health at risk:

‘[… *N*]ot comfortable to test at home […] worried about making mistakes and to have inaccurate readings …’ (P05, 32 years, 5 Sept 2018)

‘… I don’t understand the manner of self-monitoring because I am sure that I won’t be able to check myself why?’ (P13, 43 years, 10 Sept 2018)

Though the majority of participants showed openness to SMBP, owning the BP measuring machine is inconceivable because participants cannot afford to buy for themselves. Lack of confidence amongst few participants to use the BP machine as well as insufficient knowledge on the subject of SMBP was notable.

### Theme 4: Benefits of using self-monitoring of blood pressure

The SMBP at home may bring changes in the lives of patients and healthcare facilities.

**Early identification of abnormalities and seeking medical attent ion urgently:** The majority of the participants believed that SMBP pressure would make them aware of the high readings, allowing them to seek medical assistance on time. The value of monitoring BP at home was expressed as follows:

‘[… *M*]aybe your blood pressure is high then, if you can monitor it at home, then you’ll be alert and go seek help at the clinic …’ (P07, 41 years, 6 Sept 2018)

‘… I think it is right, so that I should know that my high blood is either high or down …’ (P03, 31 years, 4 Sept 2018)

‘… I must know how my status is like, my health. I must be able to take care of my health …’ (P14, 33 years, 10 Sept 2018)

‘It will help me for my life and my baby’s one. If it happens that it is high, I will be able to go see the doctor to give me medication …’ (P03, 31 years, 4 Sept 2018)

The majority of the participants believed that SMBP at home would be useful in recognising abnormal BP readings; they would then rush to the clinic or hospital to seek urgent medical care. This was expressed as follows:

‘I think that will help a lot […] you are able to see mmm, the dangers […] go to clinic for help …’ (P05, 32 years, 5 Sept 2018)

‘… I think it is right, so that I should know that my blood pressure is either high or down […] and seek medical attention urgently …’ (P03, 31 years, 4 Sept 2018)

‘… I’m supposed to go to hospital when I find it to be high when I check […] when it gets worse, I have to go to hospital …’ (P14, 33 years, 10 Sept 2018)

**Safety of the mother and the unborn baby:** The majority of the participants believed that SMBP at home will keep them and their unborn babies safer because they will immediately notice if it is high and will report it to the healthcare providers:

‘I’ll be safer. On a safe side, both myself and the baby …’ (P14, 33 years, 10 Sept 2018)

‘It will help me for my life and my baby’s one. If it happens that it is high, I will be able to go see the doctor to give me medication …’ (P03, 31 years, 4 Sept 2018)

‘… I must know how my status is like, my health … I must be able to take care of my health …’ (P14, 33 years, 10 Sept 2018)

## Discussion of key findings

Four themes were discussed. These themes were the understanding of hypertension disorders during pregnancy, openness to SMBP at home, hindrances in SMBP, and benefits of using SMBP.

The participants had a limited understanding *of hypertension disorders during* pregnancy. The majority of the participants had limited information on high BP in pregnancy because they did not get sufficient information from the media and healthcare practitioners during ANC attendance. The findings concur with the study of Akeju et al. (2016:57), which illustrate that knowledge of preeclampsia and eclampsia was limited in communities. Maputle et al. (2017:47) emphasise that there is a knowledge deficit regarding pregnancy-induced hypertension amongst pregnant women. Results from the studies of Ouasmani et al. (2018:344) and Savage and Hoho (2016:412) also confirm that pregnant women have low knowledge levels on preeclampsia or no knowledge at all (Al-Ateeq & Al-Rusaiess 2015:239). The evidence emphasises the need for more organised health educational activities at the ANC facilities.

The role of the ANC is to give dedicated high quality, culturally sensitive health education and services to families, especially pregnant women (South African Nursing Council 2014:5). Health education and advice from healthcare workers during the early ANC visit were found to have a substantial and positive impact on improving healthcare outcomes, which reduce the related complications and costs caused by late ANC initiation (Gebrekidan & Worku 2017:223).

During the research period, it was observed that there was neither any general health education nor any exercises for the women attending ANC. However, health advice was given to individuals by the attending healthcare provider, such as midwives or student medical doctors or nurses in the examination room. Pregnant women also miss the opportunity to be taught about healthy pregnancy topics because of late ANC bookings. Late or lack of ANC booking may be attributed to various reasons such as the absence or lack of support from parents and spouses, and apprehension about bewitchment because of the cultural beliefs regarding early visits to the clinic (Warrie & George 2020:1437).

A few participants had a basic knowledge of the causes and the factors that increase BP in pregnancy, such as eating unhealthy foods, including salty and fatty foods. Stress-related situations were also identified as a cause of hypertension in pregnancy. This understanding concurred with the finding of a study by Akeju et al. (2016:57), which reveal that preeclampsia is assumed to be a stress-induced condition. Rabiepour, Saboory and Abedi (2019:218) discovered a substantial association between supposed maternal stress at the time of delivery and preeclampsia.

The participants further elaborated on steps to reduce high BP, such as moderate exercising, eating healthy food, taking prescribed medication, resting and avoiding stress. The Dietary Approach to Stop Hypertension (DASH) 2020 reported that diet was beneficial in controlling BP, especially during pregnancy, to prevent the onset of preeclampsia. A diet increased intake of fruits, vegetables and lean proteins; and restricts consumption of red meat, salt, added sugars and fat (Challa, Ameer & Uppaluri 2020:29494120).

The findings from study of Abegaz et al. (2017:e5641) on pharmaceutical control of BP, refute the statement made by most participants that they are taking the medication regularly as directed by health practitioners. The study revealed that a high proportion of patients with uncontrolled hypertension was non-compliant with medication, which was common in African females.

Gupta et al. (2017:1113) confirm that it is common for patients to be non-compliant in taking anti-hypertension medication. Some participants confessed that they did not take their prescribed medication as it causes side effects such as nausea and vomiting. This is in concurrence to the findings by Israeli et al. (2018:2454), where participants did not adhere to prescribed medication due to nausea and vomiting because they did not understand the health benefits of compliance. Amongst the causes of uncontrolled hypertension, non-adherence to medication was the most common cause (Lee et al. 2019:20).

Uncontrolled hypertension can affect the growth of the placenta, affecting and restricting the nutrients and oxygen supply to the foetus. This can lead to premature delivery, low birth weight, placental separation and other problems such as eclampsia and death (Maputle et al. 2017:47). The findings of Bellad et al. (2017:e0166623) assert that with hypertension in pregnancy, seizures are associated with maternal and neonatal prematurity and deaths. Foetal growth restriction was associated with mothers who developed severe preeclampsia (Regan, Masters & Warshak 2015:322). Sharma, Shastri and Sharma (2016:67) concur that hypertensive disorders, both gestational and non-gestational, cause intrauterine growth restriction.

*The openness on SMBP at home* revealed the willingness of participants to check their BP in the comfort of their home rather than frequent clinic visits. This finding is supported by Tucker et al. (2017:442), where participants with hypertension in pregnancy were eager to participate in SMBP. Hodgkinson et al. (2014:6616) testify that SMBP is more acceptable to pregnant women compared to recurrent clinic visits or hospitalisation. Pregnant women preferred self-monitoring at home to the clinic and ambulatory measurements (Walshaw & McManus 2017:87).

However, some patients were still reluctant to monitor BP at home because of lack of knowledge and self-confidence, as they feared making mistakes when using the digital BP monitoring machine that is unfamiliar to them. They therefore preferred to attend the ANC to be checked by nurses to easily identify problems and receive immediate medical attention (Ringrose et al. 2017:685). Also, the study of Carter et al. (2018:919) was concerned about the validity of the methods of monitoring and the reliability of the BP machine.

Even though participants are open and willing to SMBP, some hindrances may prevent them from performing this task. Pregnant women will require the BP machine; thus, affordability and availability become a challenge, as some cannot afford to buy the machine. Berg (2018) also identified the cost of a home BP monitor as a barrier to self-monitoring – some patients cannot afford a quality BP monitor and yet, they need it. In the study by Carter et al. (2018:920), participants indicated that out-of-pocket costs might prevent patients from self-monitoring. Some of the participants in this study indicated discomfort in monitoring BP at home due to a lack of skills in using the machine. The study of Wake, Bekele and Tuji (2020:13) also found a lack of knowledge and confidence in operating the BP machine at home. Training is required for using the BP monitor before checking BP at home.

The outcomes of the BP measurement at home may make some participants anxious because they fear being scolded by the nurses when their BP readings are high and for being accused of having eaten the wrong food and not taking medication as prescribed. George and MacDonald (2015:95) suggest that home BP monitoring might induce anxiety and excessive monitoring due to fear or concerns that their BP could be high (Harvard Health Publishing 2018). Excessive anxiety and stress can result in uncontrolled BP during pregnancy (Ghoghre 2016:22; Kordi et al. 2016:814).

*The SMBP is valued* as it can assist preeclampsia patients in identifying abnormalities and seeking medical attention. Frequent ANC visits to check BP may be minimised (Tucker et al. 2017:442; Walshaw & McManus 2017:87). Preeclampsia patients would be empowered to take charge of their health and that of their unborn babies. They will be able to know the status of their BP measurements, whether normal or abnormal. Hinton et al. (2017:427) and Tucker et al. (2017:442) suggest that SMBP in pregnancy is feasible and that it is beneficial in the early detection of gestational hypertensive disorders, enabling patients to seek medical intervention timeously. Uncontrolled or untreated BP during pregnancy may increase maternal and neonatal mortality or morbidity rate. Similar findings were reported in the studies of Mol et al. (2016:999) and Saxena, Bava and Nandanwar (2016:2171), which confirmed that if high BP is left untreated, it is dangerous for both the mother and the child.

### Strength and limitations

The study incorporated pregnant women of a variety of ages, parity, social conditions, literacy and past experiences including those with a preeclampsia history and other pregnancy-related illness. The evidence obtained will help in addressing the knowledge gaps regarding preeclampsia and the challenges that could inhibit patients from SMBP. It would empower pregnant women in caring for themselves, thus reducing the need for frequent visits to the ANC for BP check-ups. Health facilities will have to deal with fewer attendees, in compliance with the caronavirus disease 2019 (COVID-19) guidelines. This study provides empirical evidence of the limited knowledge based on lack of health education in ANC and could thus enhance the development of health policies and health education strategies in ANCs. Participants were all Africans, only one participant obtained a post-school qualification, as such the findings may not be representative of the broader population of women with higher education levels. Qualitative research was used, and participants were purposively sampled. The study was conducted in just one public health facility in Gauteng province, in South Africa. The findings, therefore, cannot be generalised to all the pregnant women with preeclampsia in private health facilities or those in other provinces and countries.

### Recommendations

Self-monitoring of BP was well received and accepted by preeclampsia pregnant women who participated in the study. It is therefore recommended that the Department of Health introduces the use of BP machines for preeclampsia patients. Due to the unaffordability and unavailability of the BP machine, it is recommended that hospitals and clinics rendering ANC provide patients with preeclampsia and those who are at high risk, with reliable and affordable BP equipment to do self-monitoring at home. Healthcare institutions and practitioners, especially midwifery specialists, should provide health education programmes that will be monitored and measured for compliance to ensure that quality outcomes can be accurately reported.

Future studies on SMBP should be conducted using different designs, including more facilities and patients in public and public institutions in South Africa to explore the feasibility, attitudes of preeclampsia patients in other provinces as well as in the private sectors.

## Conclusion

This study displayed the participants’ interest in and openness to SMBP although they acknowledged that there were both challenges and benefits. A significant knowledge deficit was observed amongst pregnant women regarding hypertension in pregnancy even though they did have some knowledge about its causes and complications. The study also underscored the need to revisit the maternal health policies to pay special attention to health education programmes within maternal care in order to revise and update the content. Low knowledge in maternal care can be a result of a variety of reasons, which could be addressed by midwifery specialists and nurses through health promotion and prevention competencies. During the COVID-19 pandemic when the country’s regulations promote social distancing and prohibits large crowds (DoH 2020:15), SMBP is perceived to be an intervention that can be of value in reducing number of patients visiting hospitals and clinics for maternal care.
